# Mendelian randomization reveals the causal association between gout and hearing impairment in older adults

**DOI:** 10.1097/MD.0000000000038259

**Published:** 2024-05-31

**Authors:** Xiaopeng Fu, Xin Zhao

**Affiliations:** a College of Traditional Chinese Medicine, Fujian University of Traditional Chinese Medicine, Fuzhou, Fujian, China; b Beijing Chaoyang District Center for Disease Prevention and Control, Beijing, China.

**Keywords:** age-related hearing impairment (ARHL), causality effects, gout, Mendelian randomization, urate

## Abstract

With the global aging trend escalating, the holistic well-being of the elderly has become a paramount concern within public health. Traditional observational studies often struggle with confounding factors and establishing causality, leaving the relationship between age-related hearing loss (ARHL) and gout largely unexplored. Employing bidirectional two-sample Mendelian randomization (MR) analysis, this investigation elucidated the genetic underpinnings associated with age-related hearing impairment, gout, and urate levels within the IEU Open-GWAS database, thereby uncovering potential causal connections that underlie the intricate interplay between gout, serum urate concentrations, and auditory decline in the geriatric demographic. In the forward MR phase, a cohort of 30 single nucleotide polymorphisms was leveraged to dissect the causal dynamics between ARHL and both gout and urate concentrations. Conversely, in the reverse MR phase, gout and urate levels were posited as the exposome to delineate their impact on hearing acuity, employing an array of models for rigorous validation and sensitivity scrutiny. In the forward MR analysis, a statistically significant correlation was discerned between ARHL and gout (*P* = .003, odds ratio = 1.01, 95% confidence interval: 1.00–1.02), alongside a notable association with serum urate levels (*P* = .031, odds ratio = 1.39, 95% confidence interval: 1.03–1.88), intimating that ARHL could potentially influence the incidence of gout and urate concentrations. Conversely, the reverse MR investigation revealed that neither gout nor serum urate levels exerted significant impact on auditory degradation (*P* > .05), insinuating that these factors might not predominantly contribute to hearing loss. Sensitivity analyses concurred with this inference. This study enriches the comprehension of geriatric health intricacies and unveils that ARHL potentially influences gout and serum urate concentrations. This suggests that monitoring ARHL may play a crucial role in the early identification and management of gout and hyperuricemia, potentially contributing to a comprehensive approach to improving geriatric health outcomes.

## 1. Introduction

Gout, an inflammatory ailment precipitated by the deposition of monosodium urate (MSU) crystals within joint cavities,^[1]^ manifests acutely as nocturnal episodes of excruciating, knife-like pain, significantly debilitating the patient’s quality of life and escalating the economic encumbrance.^[2]^ As a global disease, gout exhibits geographical variations in prevalence, which is on an overall upward trend worldwide.^[3]^ Research indicates that in Australia, the prevalence of gout reaches as high as 6.8%.^[4]^ Moreover, the age of onset is trending younger, making the occurrence of adolescent gout increasingly common.^[5]^ Elevations and perturbations in urate levels constitute the principal etiological factors of gout.^[6]^ Urate metabolism encompasses a multifaceted system, primarily involving its production and excretion via renal and intestinal pathways. Emerging research posits^[7]^ that even marginal variations in bodily urate concentrations may confer a spectrum of salutary effects, transcending its traditional dismissal as mere metabolic detritus.^[8]^ This has led some scholars to advocate for the prognostic and therapeutic utility of urate in the context of neurodegenerative diseases.^[9]^ Age-related hearing loss (ARHL) represents the predominant auditory deficit among the elderly, afflicting approximately one-third of individuals aged 65 and over.^[10]^ ARHL can precipitate a decline in occupational productivity, engender social isolation, and catalyze depression and other psychological disturbances. Research delineates distinct associations among hearing loss, gout, and serum urate levels.^[11,12]^ Nonetheless, the elucidation of these relationships through conventional epidemiological approaches is obfuscated by potential confounders, including pain, comorbidities, and issues of reverse causality.^[13]^ Mendelian randomization (MR) embodies a genetic instrumental approach to causal inference. Its foundational premise leverages the effects of genotypes, assigned at random, on phenotypes to discern the influence of biological variables on disease manifestation. Ingeniously designed to circumvent the prevalent confounding factors and reverse causality dilemmas inherent in conventional observational studies, MR furnishes a robust framework for the ascertainment of causality. Employing a bidirectional two-sample Mendelian randomization methodology, this investigation delves into the causal nexus linking ARHL, gout, and serum urate concentrations, thereby furnishing substantiated evidence.

## 2. Materials and methods

### 2.1. Study design

**Figure 1.**
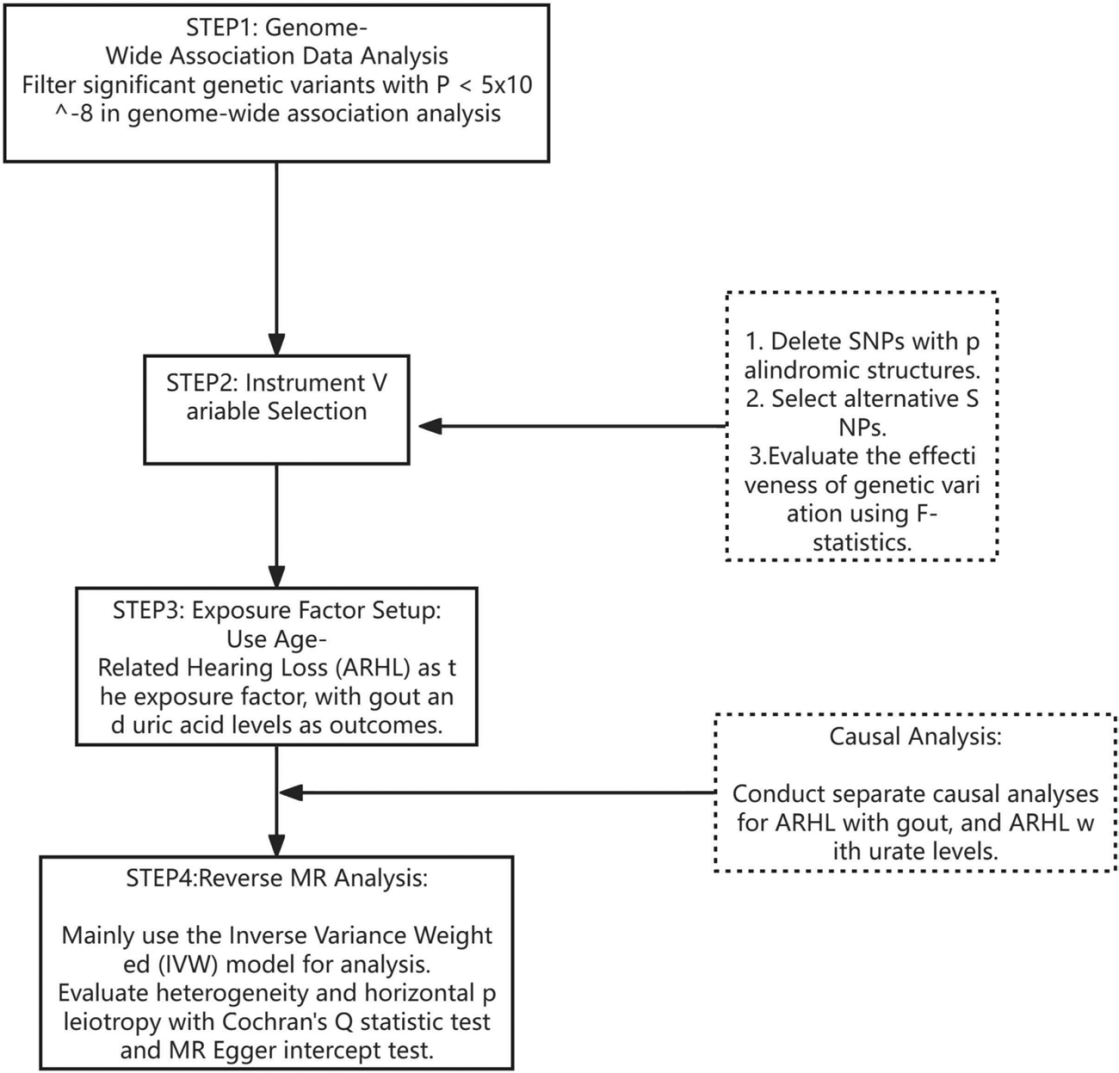
Flowchart of Mendelian randomization experiment on ARHL, gout, and urate. ARHL = age-related hearing loss.

Mendelian randomization harnesses the inherent randomness of genetic instruments to address endogeneity concerns and facilitate instrumental variable analysis.^[14]^ For genetic variations to serve as effective instruments in deducing causal relationships, they must satisfy 3 critical criteria: 1. The genetic variation is associated with the exposure; 2. It is not linked to the outcome via confounding variables; 3. It influences the outcome solely through its indirect connection with the exposure. This investigation employs a bidirectional two-sample Mendelian randomization approach to scrutinize the causal dynamics between hearing impairment and gout, alongside the relationship between hearing impairment and serum urate levels. Through this methodological framework, we aim to elucidate the ramifications of hearing impairment on gout and serum urate levels more comprehensively, leveraging the unique attributes of Mendelian randomization to mitigate potential endogeneity, thus enhancing the credibility of causal assertions, as depicted in Figure 1.

### 2.2. Genetic associations with age-related hearing impairment

The aggregate statistics for ARHL emanated from a comprehensive meta-analysis,^[15]^ analyzing GWAS datasets pertaining to 4 auditory traits collated by the UK Biobank, involving 330,759 participants. This analysis delineated 31 loci significantly correlated with self-reported auditory deficits, amongst which 23 were previously identified, and 8 emerged as novel findings (*P* < 5 × 10^−8^). The UK Biobank, an expansive biomedical investigation, amasses biological specimens, such as blood and urine, alongside extensive clinical and survey data from a vast cohort, endeavoring to meticulously examine health outcomes, genetic predispositions, and the impacts of environmental factors on phenotypic expressions.^[16]^ The meta-analysis integrated 4 GWAS summaries, all derived from self-reported survey responses probing the participants’ ability to discern conversations against background noise (e.g., TV, radio, children at play), challenges with hearing acuity, habitual use of hearing aids, and the occurrence of persistent auditory sensations like ringing or buzzing in the ears or head, lasting over 5 minutes (subsequently referred to as tinnitus, with the most inheritable response being “yes, now most or all of the time”). These inquiries served as proxies for diagnosing symptoms of hearing impairment.

The genetic insights pertinent to ARHL harnessed in this research are accessible through the IEU OpenGWAS project database (https://gwas.mrcieu.ac.uk/), catalogued under the GWAS ID “ebi-a-GCST90012115.” This MR study was conducted using publicly available studies or shared datasets, thus it did not require additional ethical statements or consent, which includes the data on urate and gout discussed below (see Table S1, Supplemental Digital Content, http://links.lww.com/MD/M596 for detailed data).

### 2.3. Genetic associations with gout and urate

#### 2.3.1. Genetic associations with gout

The summary statistics for gout are derived from self-reports on gout within the UK Biobank, as part of the non-cancer illness dataset.^[17]^ The dataset includes 6543 cases and 456,390 controls, totaling 462,933 individuals of European ancestry. Summary statistics are available in the IEU OpenGwas project database, with the GWAS ID of ukb-b-13251.

#### 2.3.2. Genetic associations with urate

The dataset for urate^[18]^ comes from a GWAS conducted by the global urate genetics consortium, which includes data from over 140,000 individuals of European ancestry. The study identified 28 genome-wide significant loci associated with serum urate concentrations. Summary statistics are available in the IEU OpenGwas project database, with the GWAS ID of “ieu-a-1055.”

### 2.4. Selection of instrumental variables

To conduct the instrumental variable analysis, we extracted information on ARHL, gout, and urate levels from the summary statistics of different genetic association studies (GWAS). Considering the biological link between gout and urate levels, we hypothesized that MTGA might indirectly influence the incidence of gout through its effect on urate levels. In the IV extraction phase, we employed a stringent clumping threshold (R^2^ < 0.001, kb = 10,000) to exclude single nucleotide polymorphisms (SNPs) with residual linkage disequilibrium (LD) within a specific window. The strength of the instrumental variables was assessed using the F-statistic to avoid the “weak instrument risk.” An F-value < 10 typically indicates a weak instrument bias.^[19]^ Moreover, we calculated R2, and when no SNP associated with our exposure of interest was found in the summary statistics of the results, we used appropriate proxy SNPs (R^2^ > 0.8). SNPs with palindromic structures were excluded during the analysis to ensure the robustness of the instrumental variables in genetic variation and causal inference.

### 2.5. Mendelian randomization analysis and sensitivity analysis

This study utilizes 5 different statistical methods^[20]^ to assess the bidirectional causal relationships between ARHL and both gout and serum urate levels via MR analysis. The inverse variance weighted (IVW) method, inspired by meta-analysis, is introduced as a statistical method to reduce variance through a weighted average approach,^[21]^ employing the inverse variance weighted method as the primary analytical approach. Sensitivity analysis of the IVW method results is conducted to verify the robustness of the study findings, using Cochran Q statistical test^[22]^ to assess heterogeneity and funnel plots to evaluate the results of the MR analysis.^[23]^ For genetic pleiotropy, the MR Pleiotropy Residual Sum and Outlier method, as well as evaluation and correction for multidirectional outliers, are conducted using the R software’s TwoSample MR package. Leave-one-out analysis for individual SNPs is performed and forest plots are drawn (see Content S2, Supplemental Digital Content, http://links.lww.com/MD/M597).

All Mendelian randomization (MR) analyses and sensitivity tests were completed using the TwoSampleMR package in R software (version 4.2.2), with funnel plots and forest plots drawn using the ggplot2 package. This ensures consistency and repeatability in the analyses.

## 3. Results

### 3.1. Selection of instrumental variables and MR analysis

**Figure 2.**
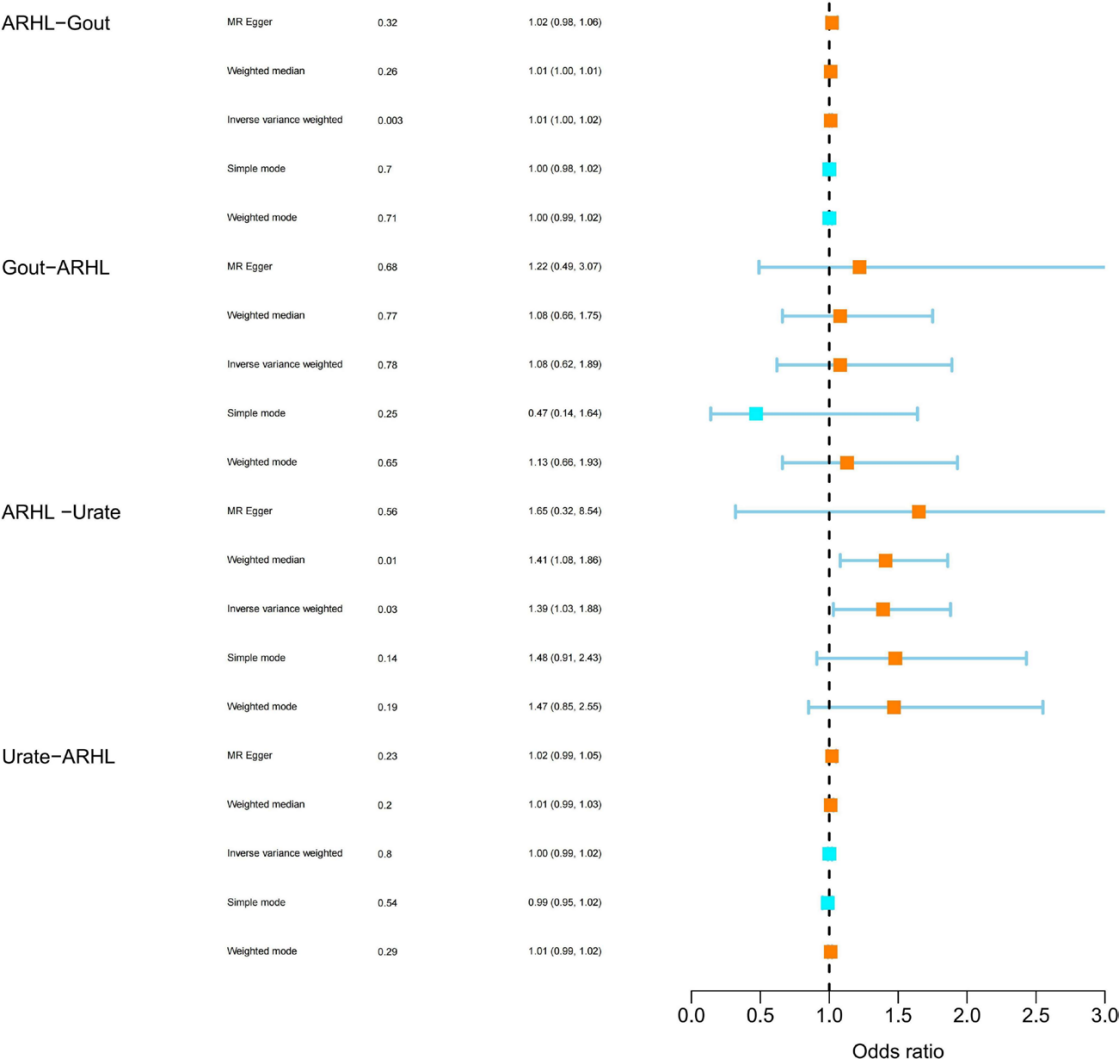
The Mendelian randomization forest plot depicts a causal relationship between gout and auditory impairment. The horizontal lines in the forest plot represent the confidence intervals for each study. Employing various Mendelian randomization methods, we comprehensively explored the causal nexus between gout and auditory impairment.

As illustrated in Figure 2 and Table 1, we selected genetic variants with significant differences at the level of *P* < 5 × 10^−8^ from the summary data of the genome-wide association analysis of MTGA with gout and urate levels. After excluding SNPs with palindromic structures and selecting alternative SNPs, in the forward MR stage, 30 SNPs were chosen as initial instrumental variables for ARHL. In the MR analysis of hearing difficulty with gout, 28 SNPs were ultimately included for MR analysis. After assessment using the F-statistic, with an average F value = 43.76 > 10, Cochran Q statistical test indicated no heterogeneity (*P* > .05). The MR Egger intercept test suggested no significant horizontal pleiotropy; in the MR analysis of hearing difficulty with urate levels, 16 SNPs were finally selected for analysis. Cochran Q statistical test indicated heterogeneity, but no horizontal pleiotropy was found after testing for horizontal pleiotropy (*P* > .05). Therefore, a random IVW model method was chosen for analysis, along with the weighted median method for reference. In the reverse MR phase, when gout was considered as the exposure factor, 23 SNPs were selected as initial instrumental variables. After deleting palindromic sequences and selecting alternative SNPs, a total of 22 SNP sites were included for further analysis. Upon testing the F-value, F = 130.15 > 10, Cochran Q statistical test indicated heterogeneity, choosing a random IVW effect model for analysis. The horizontal pleiotropy test found no horizontal pleiotropy; after treating urate levels as the exposure factor, 27 SNPs were extracted as preliminary instrumental variables. After screening, a total of 26 were included, F = 155.20 > 10, Cochran Q statistical test indicated heterogeneity, choosing an IVW random effects model for analysis, with horizontal pleiotropy testing revealing no horizontal pleiotropy.

**Table 1:** Causal Inference of ARHL in bidirectional two-sample Mendelian randomization with gout and uric acid levels.

Exposure	Outcome	SNPs(n)	F-STAT	Cochran Q P-value	MR-Egger pleiotropy test P-value	Methods	OR (95%CI)	*P*-val
ARHL	Gout	27	43.76	0.18	0.614	MR Egger	1.02 (0.98, 1.06)	.32
						Weighted median	1.01 (1.00, 1.01)	.26
						Inverse variance weighted	1.01 (1.00, 1.02)	.003
						Simple mode	1.00 (0.98, 1.02)	.70
						Weighted mode	1.00 (0.99, 1.02)	.71
Gout	Age-related hearing impairment (ARHL)	19	130.15	<0.01	0.75	MR Egger	1.22 (0.49, 3.07)	.68
						Weighted median	1.08 (0.66, 1.75)	.77
						Inverse variance weighted	1.08 (0.62, 1.89)	.78
						Simple mode	0.47 (0.14, 1.64)	.25
						Weighted mode	1.13 (0.66, 1.93)	.65
ARHL	Urate	16	43.76	<0.01	0.84	MR Egger	1.65 (0.32, 8.54)	.56
						Weighted median	1.41 (1.08, 1.86)	.01
						Inverse variance weighted	1.39 (1.03, 1.88)	.03
						Simple mode	1.48 (0.91, 2.43)	.14
						Weighted mode	1.47 (0.85, 2.55)	.19
Urate	Age-related hearing impairment (ARHL)	24	155.20	0.04	0.21	MR Egger	1.02 (0.99, 1.05)	.23
						Weighted median	1.01 (0.99, 1.03)	.20
						Inverse variance weighted	1.00 (0.99, 1.02)	.80
						Simple mode	0.99 (0.95, 1.02)	.54
						Weighted mode	1.01 (0.99, 1.02)	.29

ARHL: age-related hearing loss; SNP: single nucleotide polymorphism.

### 3.2. Causality of ARHL with gout and urate

#### 3.2.1. Causality of ARHL and gout

In the forward phase, with ARHL as the exposure and gout as the outcome, the results indicated a significant association in the inverse variance weighted method (*P* = .003, odds ratio (OR) = 1.01, 95% confidence interval (CI): 1.00–1.02). This suggests that hearing loss may have a certain impact on the development of gout. Although the *P*-values of the other 4 methods did not reach the level of significance, their consistent direction further strengthens the possibility of an association between hearing difficulty and gout. In the reverse MR phase, with the presence of gout as the exposure and hearing difficulty as the outcome, the analysis results from the reverse Mendelian randomization (MR) phase showed no significant causal association across various MR methods (*P*-value > 0.05). Furthermore, the confidence interval includes 1, indicating no evident reverse causal relationship. Therefore, based on the results of the reverse MR study, it can be concluded that there is no reverse causal relationship, that is, gout is not considered a major factor leading to hearing difficulty as shown in Table 1 and Figure 2.

#### 3.2.2. Causality of ARHL and urate levels

In the forward MR phase, with ARHL as the exposure and serum urate levels as the outcome, the results demonstrated a significant association in the inverse variance weighted method (*P* = .031, OR = 1.39, 95% CI = 1.031.88). Similar results were observed in the weighted median method (*P* = .013, OR = 1.41, 95% CI = 1.031.88). Although the remaining 3 model methods did not show statistical significance, their directions were consistent. In the reverse MR phase, with serum urate levels as the exposure factor, none of the 5 model methods showed a significant association (*P*-value > 0.05), indicating that serum urate levels are not considered a major factor leading to hearing loss, as shown in Table 1 and Figure 2.

## 4. Discussion

As a metabolic disorder, the etiology of gout is multifaceted, with hyperuricemia (HA)-induced urate deposition pinpointed as the primary culprit.^[24]^ Such accumulation not only precipitates acute arthritic episodes but may also culminate in chronic arthropathy and tophaceous formations.^[25]^ Moreover, HA exhibits a strong correlation with metabolic syndrome and cardiovascular pathologies, amplifying the holistic health jeopardy faced by patients. Thus, gout transcends its identity as a mere articulatory ailment, representing a systemic condition engendered by overarching metabolic dysregulations.^[26]^ Conversely, ARHL has escalated into a significant public health concern alongside the burgeoning trend of demographic aging, with its prevalence on the rise.^[27]^ The physiological hallmark of ARHL is the senescence of sensory hair cells and auditory nerve fibers within the inner ear.^[28]^ This degeneration precipitates a diminution in auditory receptors and neuronal damage, impairing the detection of high-frequency sounds.^[29]^ Furthermore, psychosocial dimensions, encompassing the cognitive and psychological ramifications of auditory deficits, play a pivotal role.^[30]^ Issues such as social isolation and depression, prevalent among the senior demographic,^[31]^ are intricately linked with hearing loss, underscoring the criticality of mental well-being in the context of ARHL.^[32]^ However, traditional observational research is beleaguered by methodological hurdles,^[33]^ including the ubiquity of confounding variables that obfuscate the exclusion of external perturbations. Statistical rectifications seldom neutralize their influence entirely,^[34]^ thereby stymieing the elucidation of causative links. Additionally, the potential for reverse causality complicates the analysis, as it may introduce bi-directional influences between the exposure and outcome, confusing the direction of causality.^[35]^ Mendelian randomization (MR) offers a salient remedy to these impediments, elucidating causal dynamics with enhanced clarity. In this investigation’s forward MR phase, wherein ARHL was posited as the exposure and gout as the consequent condition, a notable causal association was discerned. While alternative methodologies did not achieve statistical significance, their uniform trajectory lends credence to the nexus between auditory decline and gout. Analogous outcomes were recorded with urate concentrations as the dependent variable. The symbiotic relationship between ARHL, gout, and urate levels intimates that advancing age predisposes individuals to both auditory degradation and uric acid accrual, potentially exacerbating gout’s development or progression. The subtlety of urate elevation often escapes detection, with those afflicted by asymptomatic HA typically reporting no discernible discomfort. Conversely, hearing loss, by impinging on perceptual and communicative faculties, is readily identifiable by either the sufferer or the clinician. This suggests a paradigm wherein the clinical evaluation of elderly patients, particularly those manifesting hearing impairment, warrants a more holistic approach. Our investigation is constrained by several pivotal limitations. Foremost, the reliance on a public database precludes access to granular clinical data, thereby curtailing our capacity for nuanced analyses, such as subgroup exploration. Compounded by the restrictions of publicly available datasets, the absence of specific genomic information pertinent to adolescent auditory impairment hampers our ability to dissect the nexus between age-independent hearing deficits, gout, and urate levels. It is imperative to acknowledge that the extrapolation of our findings to disparate demographics may be circumscribed, given the European origin of the GWAS data employed. This necessitates a judicious application of our conclusions to non-European populations, including those in Asia. Nonetheless, these constraints do not detract from the import of our discoveries. The elucidated association underscores a tangible linkage between ARHL, gout, and urate, spotlighting their potential convergence within physiological and pathological frameworks. Future endeavors ought to delve into the intricate relationship amongst these conditions, aiming to unearth their shared etiological pathways. Such insights promise to forge more nuanced therapeutic and preventative strategies against gout and ARHL.

## 5. Conclusions

In essence, our research delineates a causal linkage between ARHL and both gout and urate levels. While we do not find evidence of a direct causative connection from urate concentrations to the incidence of gout and ARHL, this revelation posits that ARHL may be a potential precursor to gout and urate accumulation. Vigilance towards the auditory well-being of elderly individuals can facilitate the early detection and intervention in issues pertaining to gout and HA, thereby ameliorating their overall health trajectory. These findings are anticipated to contribute positively to the management of geriatric health, providing valuable benchmarks for subsequent clinical endeavors and investigative pursuits.

## Acknowledgments

The authors appreciate the efforts of all genetics consortiums that have made their GWAS summary data publicly available.

## Author contributions

**Conceptualization:** Xiaopeng Fu.

**Formal analysis:** Xiaopeng Fu.

**Resources:** Xiaopeng Fu, Xin Zhao.

**Software:** Xiaopeng Fu.

**Supervision:** Xiaopeng Fu.

**Validation:** Xiaopeng Fu, Xin Zhao.

**Visualization:** Xiaopeng Fu, Xin Zhao.

**Writing – original draft:** Xiaopeng Fu.

**Writing – review & editing:** Xiaopeng Fu.

## Supplementary Material




