# Supplementary material for: Mendelian randomization reveals the causal association between gout and hearing impairment in older adults
Source: Medicine (Baltimore). 2024 May 31;103(22):e38259. doi: 10.1097/MD.0000000000038259 (PMC11142788; doi:10.1097/MD.0000000000038259)
Supplement: Supplementary file 2 [file medi-103-e38259-s002.docx]

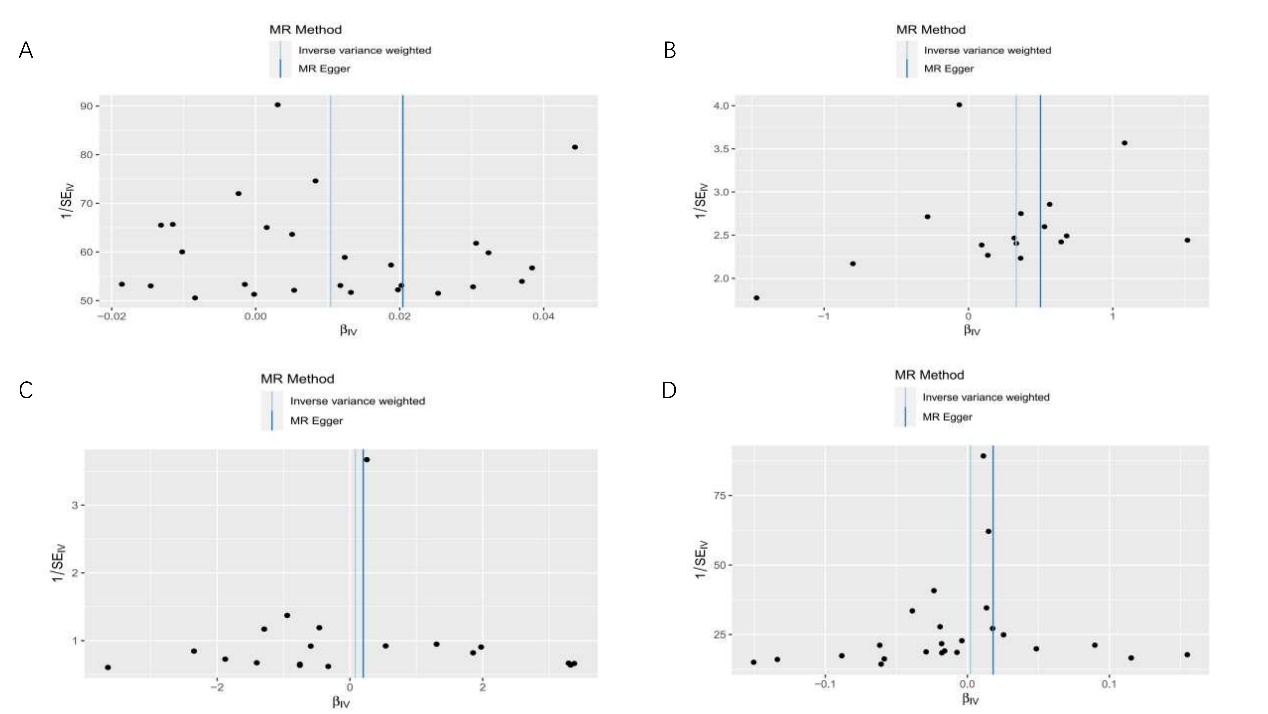


Supplementary Figure: Funnel Plot of Mendelian Randomization Analysis

Caption: This funnel plot illustrates the distribution of effect estimates from the bidirectional two-sample Mendelian randomization analysis exploring the causal relationships between age-related hearing loss (ARHL) and gout, as well as serum urate levels. The symmetry of the plot suggests the absence of substantial publication bias or heterogeneity across the selected genetic instruments used in the study. Each point represents an individual single nucleotide polymorphism (SNP) contributing to the analysis, highlighting the robustness and consistency of the causal inferences derived from our data.


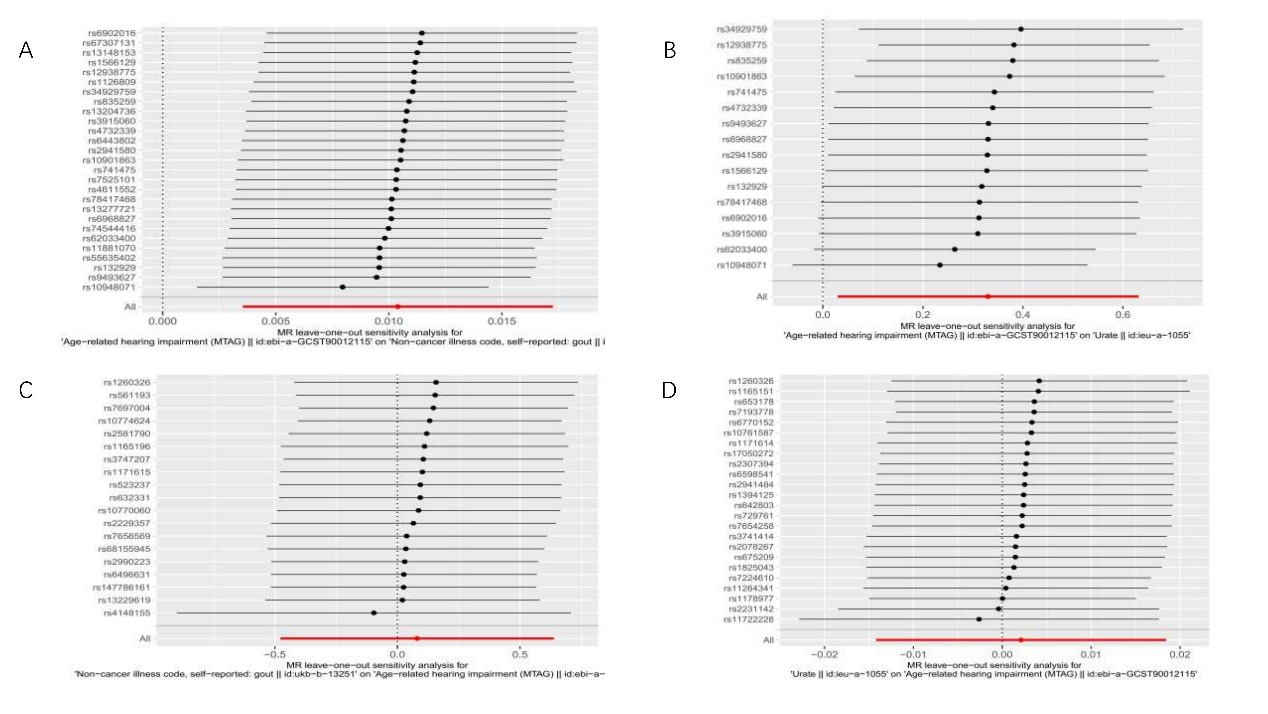


Supplementary Figure: Leave-One-Out Analysis Plot

Caption: This plot represents the Leave-One-Out analysis conducted as part of our bidirectional two-sample Mendelian randomization study. It assesses the influence of each individual single nucleotide polymorphism (SNP) on the overall robustness and stability of the causal estimates between age-related hearing loss (ARHL) and both gout and serum urate levels. The plot demonstrates that the removal of any single SNP does not significantly alter the overall causal inference, indicating that our results are not driven by any single genetic variant.


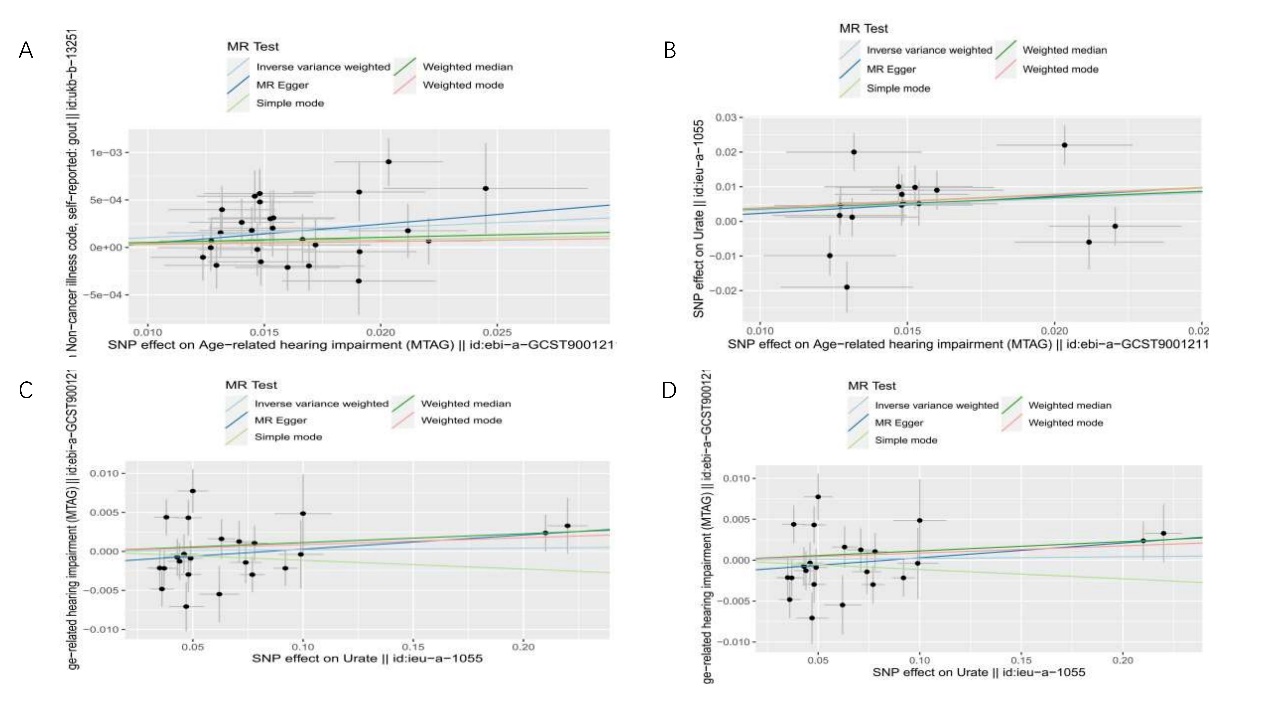


Supplementary Figure: Scatter Plot of Mendelian Randomization Analysis

Caption: This scatter plot displays the association between genetic instruments for age-related hearing loss (ARHL) and their effects on gout and serum urate levels. Each point represents a single nucleotide polymorphism (SNP) and its corresponding effect size, illustrating the genetic correlation and the strength of the causal relationship inferred. The slope of the line provides a visual representation of the estimated effect, reinforcing the genetic linkage between ARHL, gout, and serum urate concentrations as analyzed in our study.
